# Compliance to Screening Protocols for Multidrug-Resistant Microorganisms at the Emergency Departments of Two Academic Hospitals in the Dutch–German Cross-Border Region

**DOI:** 10.3390/tropicalmed6010015

**Published:** 2021-01-26

**Authors:** Lisa B. Gunnink, Donia J. Arouri, Floris E.J. Jolink, Mariëtte Lokate, Klaas de Jonge, Stefanie Kampmeier, Carolin Kreis, Michael Raschke, Mirjam Kleinjan, Jan C. ter Maaten, Alex W. Friedrich, Erik Bathoorn, Corinna Glasner

**Affiliations:** 1 Department of Medical Microbiology and Infection Control, University Medical Center Groningen, University of Groningen, Hanzeplein 1, 9713 GZ Groningen, The Netherlands; l.b.gunnink@student.rug.nl (L.B.G.); d.j.arouri@student.rug.nl (D.J.A.); f.e.j.jolink@student.rug.nl (F.E.J.J.); m.lokate@umcg.nl (M.L.); k.h.de.jonge@umcg.nl (K.d.J.); alex.friedrich@umcg.nl (A.W.F.); d.bathoorn@umcg.nl (E.B.); 2 Institute of Hygiene, University Hospital Münster, Robert-Koch-Straße 41, 48149 Münster, Germany; Stefanie.Kampmeier@ukmuenster.de; 3 Department of Trauma, Hand and Reconstructive Surgery, University Hospital Münster, Albert-Schweitzer-Campus 1, Building W1, 48149 Münster, Germany; Carolin.Kreis@ukmuenster.de (C.K.); Michael.Raschke@ukmuenster.de (M.R.); 4 Department of Emergency Medicine, University Medical Center Groningen, University of Groningen, Hanzeplein 1, 9713 GZ Groningen, The Netherlands; m.kleinjan@umcg.nl; 5 Department of Internal Medicine, Emergency Medicine, University Medical Center Groningen, University of Groningen, Hanzeplein 1, 9713 GZ Groningen, The Netherlands; j.c.ter.maaten@umcg.nl

**Keywords:** MDRO, infection prevention, screening, education, VRE, MRSA, CPE, CRE, Dutch–German cross-border region

## Abstract

Infections caused by multidrug-resistant organisms (MDROs) are associated with prolonged hospitalization and higher risk of mortality. Patients arriving in the hospital via the emergency department (ED) are screened for the presence of MDROs in compliance with the screening protocols in order to apply the correct isolation measures. In the Dutch–German border region, local hospitals apply their own screening protocols which are based upon national screening protocols. The contents of the national and local MDRO screening protocols were compared on vancomycin-resistant enterococci (VRE), methicillin-resistant *Staphylococcus aureus* (MRSA), and carbapenemase-producing and carbapenem-resistant *Enterobacteriaceae* (CPE/CRE). The practicality of the screening protocols was evaluated by performing an audit. As a result, the content of the MDRO screening protocols differed regarding risk factors for MDRO carriage, swab site, personal protective equipment, and isolation measures. The observations and questionnaires showed that the practicality was sufficient; however, the responsibility was not designated clearly and education regarding the screening protocols was deemed inappropriate. The differences between the MDRO screening protocols complicate patient care in the Dutch–German border region. Arrangements have to be made about the responsibility of the MDRO screening, and improvements are necessary concerning education regarding the MDRO screening protocols.

## 1. Introduction

The emergency department (ED) provides care to patients with acute illnesses or injuries that require immediate medical care. Thus, the ED serves as an entrance gate to the hospital for patients and the bacteria they carry with them. If the carried bacteria have acquired resistance to at least one antibiotic from three or more classes of antibiotics, they are classified as multidrug-resistant organisms (MDROs) [1,2]. The presence of a MDRO on a patient within the selective hospital environment can lead to the patient developing a (difficult-to-treat) infection, which is not only a burden to the patient themselves, but also to the hospital, as MDRO infections account for longer hospital stays, poorer patient outcomes, and increased mortality [3,4].

It is therefore important to reduce the occurrence and spread of MDROs. Several authorities around the globe, such as the European Centre for Disease Prevention and Control (ECDC) or the World Health Organization (WHO), as well as national authorities, recommend active screening for MDRO carriage [5,6,7]. Active screening allows for early detection of patients carrying MDRO, and the spread of MDROs is subsequently prevented by nursing these patients in isolation. As a result, patient safety will be improved and healthcare costs associated with MDRO infection will be reduced [8]. The strategy regarding which populations to screen differs between countries as well as between healthcare institutes within a country. In Germany, for instance, the incidence of methicillin-resistant *Staphylococcus aureus* (MRSA) is seven times higher than in the Netherlands [9]. Still, Germany and the Netherlands both advise a risk inventorization for MRSA, but healthcare institutions within the countries sometimes advise differently [10,11,12]. Moreover, depending on the MDRO species, different swab sites need to be screened. All this information regarding screening is brought together in MDRO screening protocols. MDRO screening protocols have been implemented to optimize the detection of MDROs, and for every targeted pathogen, risk groups and swab sites have been identified. MDRO screening protocols are often created by national healthcare institutions. In the Netherlands, the ‘Working Party on Infection Prevention’ (in Dutch: Werkgroep Infectie Preventie, WIP) and in Germany, the ‘Commission for Hospital Hygiene and Infection Prevention’ (in German: Kommission für Krankenhaushygiene und Infektionsprävention, KRINKO) have provided the content of the national protocols [11,12]. The MDRO screening protocols from the WIP and KRINKO are created as a minimum standard. Hospitals adjust the recommendations in these protocols for local application. In particular, in university hospitals with a large population of immune-compromised patients, screening protocols may be more extensive.

However, for the successful implementation of MDRO screening protocols at the local level, it is important that the provided information is clear and easily accessible, time for the implementation is allocated and that all healthcare professionals are educated and trained. This implementation could be challenging on an ED where the working environment is often more stressful than on a general ward with less time for MDRO risk assessment. For a screening swab to work technically, it is important that the sampling technique and choice of swabs is correct [13], and thus, healthcare professionals need to be educated in the method of sampling. Next, it is important that healthcare professionals know the responsible person or profession for taking the samples and sending them to the laboratory. As this knowledge is achieved by education, it is crucial that education is focused on the correct implementation of protocols and on maintaining compliance to these protocols. This means that the theory behind the protocols should be explained to healthcare professionals in order to value and identify why a certain protocol was developed and why it is important to follow the exact steps described in the protocols. In addition, in order to successfully and efficiently educate these healthcare professionals, the goals, professional environment, and organization should be considered and evaluated in order to facilitate a well-operating learning environment which is crucial for applying education in practice [14,15]. Finally, personal motivation, capability, and opportunity must be considered and should be promoted to ensure optimal personal development [16,17].

It is not only important that within a department or hospital, healthcare professionals know their MDRO screening protocol, but it is also crucial that they are able to communicate the results and consequences with each other. Communication about MDROs might be even more challenging when it comes to healthcare within a cross-border region. Since 2011, European citizens have had the right to be treated in any European country [18], thus patient transfers between hospitals in a cross-border region have since then been enabled and implemented [19]. Differences in MDRO classifications and screening protocols differ, for instance, between the Netherlands and Germany. This difference complicates patient care and communication between hospitals [20]. This study compared the Dutch and German national screening protocols from the WIP and KRINKO, and the locally adapted protocols from two academic hospitals within the Dutch–German cross-border region, the Dutch University Medical Center Groningen (UMCG) and the German University Hospital in Münster (UKM) for the advice on handling suspected MRSA, vancomycin-resistant enterococci (VRE), and carbapenemase-producing and carbapenem-resistant *Enterobacteriaceae* (CPE/CRE) colonization or infection. Subsequently, differences in compliance, responsibilities, and educational levels were assessed as perceived by the healthcare professionals at the participating EDs. The results of this study provide insights that are useful for the implementation of screening protocols, optimization of compliance to these protocols, and education for healthcare professionals.

## 2. Materials and Methods

### 2.1. Comparison of MDRO Screening Protocols

The contents of the MDRO screening protocols of the WIP, KRINKO, UMCG and UKM were analyzed [11,12]. The MDRO screening protocols of the UMCG and the UKM can be viewed upon inquiry. The protocol comparison included the following MDROs: VRE, MRSA, and CPE/CRE. Of note, these MDROs were chosen for comparison because the definition of these MDROs are reasonably similar between the Netherlands and Germany and this selection is not based on clinical importance [12,21,22,23,24,25].

The following variables were extracted from the protocols: the risk factors that are employed to define an MDRO risk population; the swab sites that should be sampled, the personal protective equipment (PPE) that is required, and the isolation measures that should be applied.

### 2.2. Assessment of Protocol Practicality and Compliance

In order to assess the practicality and the healthcare professionals’ compliance with the protocol, observation forms and surveys were filled out at the EDs of the UMCG and the UKM in April 2019. The observation forms were filled out prior to the surveys. The general ED of the UMCG and the trauma surgical ED from the UKM participated in this study. Participants included ED doctors, nurses, and students who were in training to become a doctor or nurse. The data analysis was performed by chi-square tests and Fisher’s exact test to assess differences between the two EDs.

### 2.3. Audit and Surveys

For the audit, observation forms were created. The items of these forms were based on the results of the protocol comparisons, and were in line with the questions from the surveys. The observation forms were filled out by the researchers during the audit at the UMCG and UKM, which took place on three consecutive days in each hospital. During the audit, the researchers observed consultations at the ED and focus was placed upon risk assessment, use of PPEs by healthcare professionals, arrangement of the MRSA isolation room and swab sampling and dispatch. The observation forms were not shown to the ED healthcare professionals. The Dutch version of the observation forms is appended to this study as Supplementary File S1.

The questions in the surveys were designed using the literature and information provided by experts from both the Departments of Medical Microbiology and Infection Prevention of the UMCG and UKM [26,27,28]. The answer options were shown in a five-level Likert-scale with the addition of “not applicable”. The survey contained questions concerning the profession of the participant; performing a risk assessment; ordering and performing swabs; isolation measures; compliance to the MDRO protocol and the healthcare professional’s opinion. Both the Dutch and the German version of the survey have been appended as Supplementary File S2.

## 3. Results

### 3.1. Comparison of MDRO Screening Protocols

The MDRO screening protocols from the WIP, KRINKO, UMCG and UKM were compared to investigate differences and similarities in order to facilitate healthcare professionals involved in cross-border healthcare. The risk factors mentioned in these four MDRO screening protocols were generally similar, especially with regard to high-risk groups including livestock-related occupations and hospitalization abroad. Differences were noted regarding previous admissions to national hospitals: the KRINKO and the UMCG advised screening a patient when they were recently admitted to another national hospital, whereas the WIP advised only screening when a patient is transferred from a hospital with an MDRO-outbreak on the ward. The UKM screened every patient that was admitted to the hospital for MRSA, and only screened for VRE in units with patients at risk and CPE/CRE in the case of an international hospital medical history. Considering admission and treatment in a foreign hospital, all four protocols advised screening for MDRO; however, the use of time frames varied. The UKM did not use any time frame, while the WIP, KRINKO, and UMCG further specified the length of stay and the number of months ago since the last admission (Table 1).

When an indication for MDRO screening is present, a swab needs to be taken from the patient and subsequently processed in order to confirm the presence/absence of any MDRO. The swab sites are dependent on the MDRO species that were expected according to the patient’s risk factors. The swab sites were compared for VRE, MRSA, and CPE/CRE among the MDRO screening protocols to identify differences in advised swab sites. The swab site comparison showed that all four protocols advised at least one unified swab site per MDRO. In the Dutch protocols, faecal samples were considered just as appropriate as rectal samples for VRE and CPE/CRE screening. Considering the MRSA screening, all protocols advised at least the use of a nasal swab, a throat swab, as well as a wound swab (Table 2). For VRE and CPE/CRE, UKM advised additional sampling of wounds and previously positive sites.

Healthcare professionals protect themselves from contamination and transmission of MDROs when they come in contact with a patient suspected of MDRO colonization or infection. This protection is offered by the use of PPE. The advised selection of PPE is dependent on the suspected MDRO species.

The protocol comparison of PPE showed that the protocols shared the same advice for using an apron, a surgical mouth-nose mask, and gloves for healthcare professionals who were in contact with an MRSA-positive patient, but the hair cap was only advised in both the WIP and UMCG protocols. Concerning the use of gloves, all protocols advised that gloves should always be used by healthcare professionals who are in contact with a suspected or proven MDRO-positive patient (Table 3).

### 3.2. Assessment of Protocol Practicality and Compliance

#### 3.2.1. Observation Forms

The observation forms were filled out at the ED of both hospitals by the researchers. In the UMCG, 4 and in the UKM, 16 consultations were observed. Researchers observed the healthcare professional responsible for the MDRO screening, the risk assessment performed, the donning and doffing of PPEs, swab sampling and the interior of the MRSA room.

##### Summary of Observations in the UMCG

The ED of the UMCG treated patients with urgent or immediate life-threatening conditions predominantly. Risk inventories were carried out by receptionists, nurses, and ambulance personnel. Emphasis was placed on the question about admission to a foreign hospital. A single-person MRSA room with a double-door entrance was present at the UMCG ED. During the observations, none of the observed consultations took place in the MRSA room because the results of the observed risk inventories turned out to be negative. Because of the negative risk inventories, no sample taking was witnessed.

##### Summary of Observations in the UKM

At the surgical ED in the UKM, approximately 85% of patients did not present with an immediate life-threatening condition, as many patients had a scheduled appointment to prepare for a surgical intervention planned in the upcoming days. MRSA screening, which was done by nurses, took place in roughly 90% of cases that were admitted consequently. In none of the observed consultations were patients asked about a previous admission to a foreign hospital. As a result, no CPE/CRE screening was observed. During the MRSA screening, swab samples were taken from the oral mucosa instead of the throat. An MRSA room with a single-door entrance was present. During the observed visit of an MRSA-positive patient, two doctors and one nurse entering the MRSA room did not dress up according to the protocol.

#### 3.2.2. Surveys

At the UMCG, 24 surveys were filled out by 7 doctors, 14 nurses, and 3 students, and at the UKM, 23 surveys were filled out by 9 doctors, 10 nurses, and 4 students. Healthcare professionals from both EDs were satisfied with the overall practicality of the local implemented protocol. However, they were unable to comply with the MDRO screening protocols when it came to screening every patient. Healthcare professionals from both the UMCG and UKM stated they knew when to perform MDRO screening. The healthcare professionals from the UKM knew more often how to request and send samples compared to the UMCG healthcare professionals.

Healthcare professionals from the UMCG were unsatisfied about the communication about the MDRO status and healthcare professionals from both hospitals were unsatisfied about the provided education on MDRO screening (Table 4). Students in training to become a doctor or nurse scored, on average, one level lower on the Likert scale.

Responsibility as defined in the protocols was compared to responsibility as investigated by the surveys. In the UMCG, doctors had final responsibility for the MDRO screening according to the protocol. In the UKM, no responsible healthcare professional was defined. All UMCG doctors and 88.9% of UKM doctors answered that nurses are responsible for MDRO screening. UMCG nurses mainly thought that nurses were responsible for the MDRO screening, while the majority of UKM nurses considered MDRO screening a shared responsibility of both doctors and nurses. (Table 5). Observations by the researchers showed that only nurses performed the MDRO screening at both hospitals.

## 4. Discussion

As a result of globalization and mobility, more people seek medical care abroad, and patients are transferred between different healthcare institutes within a country or across a national border. This could pose an increased risk of MDRO transmissions, and there have been calls for more harmonization regarding MDRO screening internationally, and especially in border regions [29,30,31]. In order to improve patient care and reduce MDRO occurrence in the Dutch–German border region, the aim of the study was to compare and evaluate bacterial MDRO screening protocols from the WIP, KRINKO, UMCG, and UKM.

The different aspects of the MDRO screening protocols were analyzed. Overall, for several aspects of the screening protocol, differences between the national and local version of the protocols were present. With regard to the included risk factors, differences became evident in the case of ‘previous admission to a foreign hospital’. The UMCG, for instance, screened patients up to a year after admission to a foreign hospital and directly isolated patients who had been admitted in the past two months. The UKM also screened patients up to a year after admission to a foreign hospital, but they did not further specify the length of stay. Nonetheless, it should be considered that no consensus is reached by the literature regarding the duration of possible MDRO colonization [32]. In addition, there is no consensus about which risk factors should be included for screening and isolation. For instance, well-established risk factors such as frequent antibiotic use and immune suppression are not included in the protocols described in this study. Factors such as local epidemiology, isolation capacity, resources and transmission risks within a healthcare institution should be considered in the decision of which risk factors to include in protocol, and are thus dependent on the local situation. Healthcare professionals should be aware, however, of such local differences within regional healthcare networks so that they are able to inform the patients about these different strategies.

In this study, we focused on screening protocols on the ED. Protocols for patients that are admitted to wards with highly vulnerable patients such as haematology and intensive care are often more extensive. Compliance to screening protocols for patients that are directly admitted on wards is equally important as on the ED; however, this was outside the scope of our study.

Interestingly, there are several differences not only between the two countries but most importantly between the national and local guidelines within one of the two countries with regard to PPE recommendations for VRE and CPE/CRE. The local guidelines of the UMCG and UKM are stricter compared to the national guidelines. With regard to PPE when caring for an MRSA-positive patient, the majority of the content of the protocols were the same between the Dutch and German but also between the national and local versions. Differences were observed with regard to the usage of a hair cap, which was only included in the Dutch protocols of the WIP and UMCG. Since *Staphylococcus aureus* colonizes the skin, the bacterium is commonly found around the hairline. The bacterium could then potentially be shed off from patients to healthcare professionals when no hair cap is used. However, the efficacy of the hair cap has not been proven by the scientific literature up until now [33,34].

Practicality of the protocols and the healthcare professionals’ compliance regarding MDRO screening was studied using surveys and observation forms. Healthcare professionals from both hospitals were malcontent about the provided education regarding MDRO screening. Therefore, the educational program for healthcare professionals working at the ED should be re-evaluated in order to improve compliance and adherence to MDRO screening including associated application of PPE and isolation measures [35,36]. The American Centre for Disease Control and Infection suggests providing healthcare professionals with periodic education and training on MDRO risks and prevention strategies [37]. However, as the healthcare structures differ substantially between the Netherlands and Germany [38], and as such, also between the UMCG and UKM, research is required to discover an educational approach that fits each hospital individually. To start with, more emphasis could be placed on the responsibility and division of tasks related to the screening. When the division of tasks is clearly defined for all healthcare professionals and all other personnel involved, screening can be performed more efficiently. This has also been described by the literature studying the compliance to the correct use of PPE [39], in which healthcare professionals stated that proper communication and involvement of other personnel is crucial in order to perform safe clinical practice, as well as the presence of proper role models and leaders. The latter is especially important for new healthcare professionals and students [14,40,41]. Education provided in teams could enhance the teamwork between different types of personnel in the department and educate all together. This also accounts for the students present on the ED: as they on average scored lower on the Likert-scale, they could probably benefit from extra training in MDRO screening. Nonetheless, this poses new challenges regarding prior knowledge on the subject, the diversity of the group, and the chance and opportunity for the education to be successfully implemented into practice [42]. However, improving teamwork could also contribute to an improved professional environment in which there are fewer boundaries for peer review, which has also been shown to contribute to education and compliance in clinical practice [40,43].

Additionally, the education has to be continuously re-evaluated and the training sessions could be designed as case studies in order to improve the quality of the training and subsequently improve the compliance and knowledge of healthcare professionals. These suggestions will also improve the confidence of healthcare professionals and make sure that all healthcare professionals are involved during the screening [35]. Alongside the aforementioned recommendations, studies have also shown that visual reminders placed around the department aid in adherence to guidelines by healthcare professionals [44]. Moreover, the theory behind certain guidelines, such as the screening protocols, could also help healthcare professionals to value and identify why it is important to screen according to certain protocols. This is especially important because the site of sample taking (i.e., performing the swab) could influence the result when performed at the wrong site. When the theory is explained concisely and clearly, healthcare professionals find it easier understand the reason behind these tasks, which makes it easier to apply and adhere to the protocols in clinical practice. However, the workload of healthcare professionals has to be considered when implementing or re-evaluating education [14,41]. Finally, to ensure an optimal implementation of education, the goals, professional environment, and organization should be considered and evaluated in order to facilitate a well-operating learning environment [14,15]. Furthermore, human behaviour and intrinsic motivation, i.e., personal motivation, capability, and opportunity, have to be considered while implementing educational interventions and should be promoted to ensure optimal personal development [16,17]. Together with the aforementioned division of tasks and responsibilities, screening can be performed more efficiently and effectively while the involvement of healthcare professionals is also improved [45].

One remarkable observation with regard to sample sites was done by the researchers at the UKM, where swabs to test for MRSA were taken from the oral mucosa instead of the throat and/or nose. This was rather remarkable, considering the fact that *Staphylococcus aureus* colonizes the nasopharyngeal area and not the oral mucosa [33,34]. Combining both a nasal and throat swab increases the yield from 37% (only nose swab) to 50% (nose and throat swab), and thereby improves the overall sensitivity for MRSA screening by 25,7%. More patients could have been tested as false-negative if MRSA screening was performed incorrectly. This emphasizes once again the importance of education for proper MDRO screening [35,36,46,47,48].

Several limitations were encountered during this study. First, at the German academic hospitals, the surgical ED participated in this study, whereas this hospital also has an internal medicine ED. The results might thus not represent the overall ED situation at the UKM. Additionally, in the UMCG, fewer observations were possible due to the more serious medical condition of patients. Moreover, healthcare professionals of both hospitals may have unintentionally performed the screening more often and better as they were aware of the observations (Hawthorne effect).

## 5. Conclusions

This study provides the first extensive overview of cross-border MDRO screening by including protocol comparison, surveys among healthcare professionals and observations made by the researchers. Considering globalization, cross-border collaboration regarding patient care is essential in order to control MDRO transmission. This study emphasizes the need for awareness of differences in MDRO screening protocols, and, if eligible, harmonization of these protocols. In addition, this study underlines the importance of improvement of MDRO screening education [20,29,30]. International training programs for infection prevention control specialists in which expertise and competency is shared, such as that organized by the ECCMID study group EUCIC, strengthen the harmonization in screening and isolation procedures.

## Figures and Tables

**Table 1:** Overview of MDRO risk factors and screening criteria recommendations.

	National		Hospital	
	WIP	KRINKO	UMCG	UKM
Known MDRO carrier	✓	✓	✓	✓
Known contact of MDRO carrier	✓	✓	✓	✓
Contact inside the hospital	✓	✓	✓	✓
Contact outside the hospital (household member)	✓	-	✓	-
Recent admission to another national hospital	- ^1^	✓	- ^2^	- ^3^
Recent admission to a foreign hospital	✓	✓	✓	✓ ^3,4^
Admitted <24 h, <2 mo. ago and ≥1 risk factor ^5^	✓	-	✓	-
Admitted <24 h, 2–12 mo. ago and ≥1 risk factor ^5^	-	-	✓	-
Admitted >24 h, <2 mo. ago	✓	✓	✓	-
Admitted >24 h, 2–12 mo. ago	-	✓	✓	-
Recent invasive procedure in foreign hospital	✓	-	✓	- ^3^
Native and foreign patients who dialyzed abroad	✓	✓	✓	- ^3^
Occupational exposure to pigs, cattle, or poultry ^6^	✓	✓	✓	✓
Asylum seeker, refugee, or internationally adopted child	✓	✓	✓	✓ ^3^
Persistent skin lesions	-	✓	-	-

KRINKO, Kommission für Krankenhaushygiene und Infektionsprävention; MDRO, multi-drug resistant organism; UKM, Münster University Hospital; UMCG, University Medical Center Groningen; WIP, Werkgroep Infectie Preventie. Note: Rows accentuated with green indicate that this entry was mentioned in all MDRO screening assessment protocols. ^1^ Screening is only performed in case of an MDRO outbreak at the ward from the hospital the patient is admitted from. ^2^ Every patient directly admitted from another national hospital is screened upon admission to the UMCG. ^3^ The UKM screens every hospitalised patient for MRSA, independent from whether he/she was admitted from another hospital or not. For VRE, screening is only performed in units with patients at risk, to which the ED department does not belong after internal evaluation. For CPE/CRE screening swabs from patients with an international hospital medical history (without time frame) are taken. ^4^ Admitted in the last 12 months, no length of stay specified. ^5^ Risk factors: recent invasive procedure in foreign hospital, chronic infections, persistent skin lesions and presence of abscesses. ^6^ Livestock meant for meat production.

**Table 2:** Overview of MDRO swab-site recommendations.

		Faecal	Mouth	Nose	Perineal	Rectal	Throat	Wounds
VRE	WIP	✓	-	-	-	✓	-	-
	KRINKO	✓	-	-	-	✓	-	-
	UMCG	✓	-	-	-	✓	-	-
	UKM	-	-	-	-	✓	-	✓
MRSA	WIP	-	-	✓	✓	✓	✓	✓
	KRINKO	-	-	✓	-	-	✓	✓
	UMCG	-	-	✓	✓	-	✓	✓
	UKM	-	-	✓	-	-	✓	✓
CPECRE	WIP	✓	-	-	-	✓	-	-
	KRINKO	-	✓	-	-	✓	✓	-
	UMCG	✓	-	-	-	✓	✓	-
	UKM	-	-	-	-	✓	✓	✓

CPE, carbapenemase-producing *Enterobacteriaceae*; CRE, carbapenem-resistant *Enterobacteriaceae*; KRINKO, Kommission für Krankenhaushygiene und Infektionsprävention; MDRO, multi-drug resistant organism; MRSA, methicillin-resistant *Staphylococcus aureus*; UKM, Münster University Hospital; UMCG, University Medical Center Groningen; VRE, vancomycin-resistant enterococci; WIP, Werkgroep Infectie Preventie. Note: Rows accentuated with green indicate that this entry was mentioned in all MDRO protocols.

**Table 3:** Overview of personal protective equipment recommendations for healthcare professionals.

		Apron (Long-Sleeved)	Surgical Mouth-Nose Mask	Gloves	Hair Cap
VRE	WIP	-	-	✓	-
	KRINKO	✓	-	✓	-
	UMCG	✓	-	✓	-
	UKM	✓	✓	✓	-
MRSA	WIP	✓	✓	✓	✓
	KRINKO	✓	✓	✓	-
	UMCG	✓	✓	✓	✓
	UKM	✓	✓	✓	-
CPE CRE	WIP	-	-	✓	-
	KRINKO	✓	✓	✓	-
	UMCG	✓	-	✓	-
	UKM	✓	✓	✓	-

CPE, carbapenemase-producing *Enterobacteriaceae*; CRE, carbapenem-resistant *Enterobacteriaceae*; KRINKO, Kommission für Krankenhaushygiene und Infektionsprävention; MDRO, multi-drug resistant organism; MRSA, methicillin-resistant *Staphylococcus aureus*; UKM, Münster University Hospital; UMCG, University Medical Center Groningen; VRE, vancomycin-resistant enterococci; WIP, Werkgroep Infectie Preventie. Note: Rows accentuated with green indicate that this entry was mentioned in all MDRO protocols.

**Table 4:** Survey responses of healthcare professionals for MDRO screening.

			N=	Answer Option				
				Always (%)	Very often (%)	Sometimes (%)	Rarely (%)	Never (%)
Screening	Knows when to perform screening	UMCG	24	29.2	58.3	8.3	4.2	0.0
		UKM	23	34.9	39.1	21.7	4.3	0.0
Education	Knows where to obtain screening information	UMCG	23	13.0	52.2	17.4	8.7	8.7
		UKM	23	43.5	43.5	13.0	0.0	0.0
	Is satisfied with screening education	UMCG	21	0.0	9.5	52.4	38.1	0.0
		UKM	22	4.5	9.1	59.1	18.2	9.1
Diagnostics	Knows what type of swab to choose	UMCG	23	4.4	47.8	34.8	8.7	4.3
		UKM	23	30.4	34.8	26.1	8.7	0.0
	Knows how to request laboratory diagnostics	UMCG	20	5.0	40.0	50.0	0.0	5.0
		UKM	18	61.1	16.7	11.1	0.0	11.1
	Knows how to obtain samples correctly	UMCG	23	21.8	47.8	26.1	4.3	0.0
		UKM	22	31.9	40.9	22.7	4.5	0.0
	Knows how to send samples to laboratory	UMCG	22	36.5	40.9	4.5	13.6	4.5
		UKM	20	70.0	10.0	5.0	10.0	5.0
Isolation	Isolation measures are clearly mentioned	UMCG	24	4.2	50.0	45.8	0.0	0.0
		UKM	23	30.5	47.8	17.4	4.3	0.0
	MDRO status is clearly communicated	UMCG	22	18.3	40.9	13.6	22.7	4.5
		UKM	23	17.4	60.9	13.0	8.7	0.0
Over-all	MDRO screening is doable	UMCG	21	9.5	76.2	14.3	0.0	0.0
		UKM	23	39.2	47.8	13.0	0.0	0.0

MDRO, multi-drug resistant organism; UKM, Münster University Hospital; UMCG, University Medical Center Groningen. Note: To improve table readability, answer options chosen by ≥20% of participants are accentuated in green.

**Table 5:** Survey responses of healthcare professionals for MDRO screening responsibility.

	Participants		N=	Answer Option			
				Doctor (%)	Nurse (%)	Both (%)	Unsure (%)
Responsible in guidelines	Doctor	UMCG	7	0.0	100.0	0.0	0.0
		UKM	9	0.0	88.9	11.1	0.0
	Nurse	UMCG	14	7.1	42.9	14.3	35.7
		UKM	10	20.0	30.0	50.0	0.0
Performing screening assessment	Doctor	UMCG	7	0.0	85.7	0.0	14.3
		UKM	9	0.0	100.0	0.0	0.0
	Nurse	UMCG	14	0.0	78.6	14.3	7.1
		UKM	10	0.0	90.0	10.0	0.0

MDRO, multi-drug resistant organism; UKM, Münster University Hospital; UMCG, University Medical Center Groningen. Note: To improve table readability, answer options chosen by ≥20% of participants are accentuated in green.

## Data Availability

Not applicable.

## Supplementary Materials

The following are available online at https://www.mdpi.com/2414-6366/6/1/15/s1: File S1: Surveys in Dutch and German, and File S2: Observation form as used by the researchers in Dutch.
