# Supplementary material for: Compliance to Screening Protocols for Multidrug-Resistant Microorganisms at the Emergency Departments of Two Academic Hospitals in the Dutch–German Cross-Border Region
Source: Trop Med Infect Dis. 2021 Jan 26;6(1):15. doi: 10.3390/tropicalmed6010015 (PMC7838951; doi:10.3390/tropicalmed6010015)
Supplement: Supplementary file 1 [file tropicalmed-06-00015-s001.pdf]

Tropical Medicine and Infectious Disease

MDPI

Compliance to Screening Protocols for Multidrug-Resistant Microorganisms at the Emergency Departments of Two Academic Hospitals in the Dutch–German Cross-Border Region

Lisa B. Gunnink [1,†], Donia J. Arouri [1,†], Floris E.J. Jolink [1,†], Mariëtte Lokate [1], Klaas de Jonge [1], Stefanie Kampmeier [2], Carolin Kreis [3], Michael Raschke [3], Mirjam Kleinjan [4], Jan C. ter Maaten [5], Alex W. Friedrich [1], Erik Bathoorn [1,‡] and Corinna Glasner [1,*,‡]

Supplementary File 1: Observation form in Dutch

**1** Zorgverlener is werkzaam als:	Arts / Verpleegkundige			
**2** Zorgverlener kent SEH stroomdiagram uit het hoofd (NL):	Ja / Nee			
**3** Aantal patiënten die de zorgverlener in een uur tijd ziet:	………. Patiënten			
**4** Aantal juist-uitgevoerde risico-inventarisaties in een uur:	………. Risico-inventarisaties			
	**Patiënt 1**	**Patiënt 2**	**Patiënt 3**	**Patiënt 4**
**5a** Tijdens een overdracht binnen de SEH is verteld:<br><br>a. Of de patiënt gescreend is;<br>b. Of er isolatiemaatregelen van toepassing zijn;<br>c. Of er een kweek is afgenomen;<br>d. Zo ja, wordt de BRMO dan ook genoemd?	a. Ja / Nee<br>b. Ja / Nee<br>c. Ja / Nee<br>d. Ja / Nee	a. Ja / Nee<br>b. Ja / Nee<br>c. Ja / Nee<br>d. Ja / Nee	a. Ja / Nee<br>b. Ja / Nee<br>c. Ja / Nee<br>d. Ja / Nee	a. Ja / Nee<br>b. Ja / Nee<br>c. Ja / Nee<br>d. Ja / Nee
**5b** Tijdens de overdracht naar een afdeling is verteld:<br><br>a. Of de patiënt gescreend is;<br>b. Of er isolatiemaatregelen van toepassing zijn;<br>c. Of er een kweek is afgenomen;<br>d. Zo ja, wordt de BRMO dan ook genoemd?	a. Ja / Nee<br>b. Ja / Nee<br>c. Ja / Nee<br>d. Ja / Nee	a. Ja / Nee<br>b. Ja / Nee<br>c. Ja / Nee<br>d. Ja / Nee	a. Ja / Nee<br>b. Ja / Nee<br>c. Ja / Nee<br>d. Ja / Nee	a. Ja / Nee<br>b. Ja / Nee<br>c. Ja / Nee<br>d. Ja / Nee
**6** Zorgverlener vertelt belang risico-inventarisatie aan patiënt.	Ja / Nee	Ja / Nee	Ja / Nee	Ja / Nee
**7** Zorgverlener voert risico-inventarisatie uit (Mag ook fout!)	Ja / Nee	Ja / Nee	Ja / Nee	Ja / Nee
**8** Alle vragen uit het SEH-stroomdiagram worden gesteld.	Ja / Nee	Ja / Nee	Ja / Nee	Ja / Nee
**9** Zorgverlener neemt kweken af volgens SEH-stroomdiagram.	Ja / Nee	Ja / Nee	Ja / Nee	Ja / Nee
**10** Voor welke BRMO is een kweek afgenomen?	……….	……….	……….	……….
**11** Welke kweken worden er afgenomen?	Neusgat (li)<br>Neusgat (re)<br>Keel<br>Perineum<br>Rectum/Anus	Neusgat (li)<br>Neusgat (re)<br>Keel<br>Perineum<br>Rectum/Anus	Neusgat (li)<br>Neusgat (re)<br>Keel<br>Perineum<br>Rectum/Anus	Neusgat (li)<br>Neusgat (re)<br>Keel<br>Perineum<br>Rectum/Anus

	Wonden	Wonden	Wonden	Wonden
**12** Isolatiemaatregelen<br><br>a. Zorgverlener legt patiënt in juiste isolatie.<br>b. Zorgverlener gebruikt juiste persoonlijke bescherming.<br>c. Zorgverlener gaat voorbereid de kamer in / uit.	 <br>a. Ja / Nee<br>b. Ja / Nee<br><br>c. Ja / Nee	 <br>a. Ja / Nee<br>b. Ja / Nee<br><br>c. Ja / Nee	 <br>a. Ja / Nee<br>b. Ja / Nee<br><br>c. Ja / Nee	 <br>a. Ja / Nee<br>b. Ja / Nee<br><br>c. Ja / Nee
**13** Zorgverlener weet waar de kweken heen gestuurd moeten.	Ja / Nee	Ja / Nee	Ja / Nee	Ja / Nee

Supplementary File 2: Survey in Dutch and German

Survey in Dutch

Beste zorgverlener,

Allereerst willen we u bedanken voor deelname aan deze enquête.

Deze enquête is opgesteld door drie derdejaars geneeskundestudenten van de Rijksuniversiteit Groningen. De studenten schrijven ter afsluiting van hun Bachelorfase een thesis over een onderwerp naar keuze.

Dit onderzoek gaat over de screening op Bijzonder Resistente Micro-organismen (BRMO's) op de Spoedeisende Hulp (SEH). Hierbij kijken we naar verschillende aspecten, zoals de risico-inventarisatie, het afnemen en verwerken van de kweken en eventuele vervolgmaatregelen zoals isolatie. Deze enquête is gemaakt om er achter te komen wat de ervaring van zorgverleners is met het huidige SEH: MRSA-BRMO stroomdiagram. Daarnaast wordt er gekeken op welke manier de uitvoerbaarheid van dit stroomdiagram te verbeteren is.

Onderaan de bladzijden vindt u een groen vakje met ruimte voor eventuele opmerkingen.

Nogmaals, hartelijk dank voor het deelnemen aan deze enquête.

Jasmine Arouri, Floris Jolink en Liesbeth Gunnink

1. Ik ben werkzaam als:
- o Arts (Specialist, AIOS, ANIOS)
- o Student/co-assistent
- o Verpleegkundige
- o Anders, namelijk:

Ruimte voor opmerkingen:

Risico-inventarisatie

2. Ik weet in welke situaties een risico-inventarisatie voor resistente micro-organismen uitgevoerd moet worden.
    - Altijd
    - Vaak
    - Soms
    - Zelden
    - Nooit
    - Niet van toepassing

3. Door wie moet de risico-inventarisatie worden uitgevoerd op de SEH volgens de afspraken?
    - Door een arts
    - Door een student/co-assistent
    - Door een verpleegkundige
    - Ik weet het niet

4. Door wie wordt de risico-inventarisatie daadwerkelijk uitgevoerd?
    - Door een arts
    - Door een student/co-assistent
    - Door een verpleegkundige
    - Ik weet het niet

5. Als ik vragen heb over de risico-inventarisatie, dan weet ik waar ik deze informatie kan vinden.
    - Altijd
    - Vaak
    - Soms
    - Zelden
    - Nooit
    - Niet van toepassing

6. In uw perceptie, bij welk percentage van het aantal patiënten dat wordt opgenomen, bent u in staat om de risico-inventarisatie af te nemen?
    - 0% - 25%
    - 25% - 50%
    - 50% - 75%
    - 75% - 100%

7. Wanneer de risico-inventarisatie niet wordt afgenomen door mij, dan vind ik dat:
   - Erg
   - Neutraal
   - Niet erg
   - Niet van toepassing

8. Hoe tevreden bent u over de training / het onderwijs wat betreft de risico-inventarisatie?
   - Helemaal tevreden
   - Tevreden
   - Neutraal
   - Ontevreden
   - Helemaal ontevreden
   - Niet van toepassing

Aanvragen kweken

9. Na de risico-inventarisatie weet ik precies welke kweek moet worden aangevraagd.
   - Altijd
   - Vaak
   - Soms
   - Zelden
   - Nooit
   - Niet van toepassing

10. Voor mij is het duidelijk hoe ik de kweek moet aanvragen in het EPD.
   - Altijd
   - Vaak
   - Soms
   - Zelden
   - Nooit
   - Niet van toepassing

Uitvoeren kweken

11. Als ik een kweek moet afnemen weet ik precies waar en hoe ik die moet afnemen.
   - Altijd
   - Vaak
   - Soms
   - Zelden
   - Nooit
   - Niet van toepassing

12. Nadat ik een kweek heb afgenomen, weet ik waar het heen moet worden gestuurd.
- o Altijd
- o Vaak
- o Soms
- o Zelden
- o Nooit
- o Niet van toepassing

Vervolgmaatregelen en isolatie

13. Het is duidelijk aangegeven of isolatiemaatregelen van toepassing zijn bij een bepaalde patiënt (ofwel in de kamer, ofwel in het dossier).
- o Altijd
- o Vaak
- o Soms
- o Zelden
- o Nooit
- o Niet van toepassing

14. Tijdens de overdracht wordt mij verteld welke ziekteverwekker een patiënt heeft en waarom hij daarvoor in een dergelijke isolatie ligt.
- o Altijd
- o Vaak
- o Soms
- o Zelden
- o Nooit
- o Niet van toepassing

Compliance

15. Ik vind de risico-inventarisatie uitvoerbaar.
- o Altijd
- o Vaak
- o Soms
- o Zelden
- o Nooit
- o Niet van toepassing

16. Als u de risico-inventarisatie zou moeten beoordelen, wat zou uw mening dan zijn?
- o De inventarisatie is goed
- o De inventarisatie is voldoende
- o De inventarisatie is matig
- o De inventarisatie is slecht
- o Niet van toepassing

Ruimte voor verbetering

17. Waar bent u tevreden over wat betreft de risico-inventarisatie?

Antwoord:

18. Hoe kan de risico-inventarisatie verbeterd worden volgens u?

Antwoord:

Survey in German

Sehr geehrtes Gesundheitspersonal,

wir möchten uns herzlich bei Ihnen bedanken für Ihre Bereitschaft diesen Fragebogen auszufüllen. Diese Umfrage wurde von drei Medizinstudenten der Niederländischen Universität Groningen erstellt. Die Studenten erarbeiten zum Abschluss ihrer Bachelorphase eine Bachelorarbeit nach einem Thema ihrer Wahl.

Diese Studie umfasst das Screening von multiresistenten Bakterien in der Notaufnahme. Hierbei untersuchen wir die verschiedenen Aspekte, wie die Screening, die Abnahme der Screeningabstrich und eventuell daraus resultierende Maßnahmen.

Mittels dieser Umfrage wollen wir feststellen wie das Gesundheitspersonal das derzeitige Aufnahmescreening beurteilt.

An jedem Seitenende können Sie Ihre Bemerkungen in dem grün markierten Feld anbringen. Wir bedanken uns nochmals herzlich für Ihre Teilnahme an dieser Umfrage.

Jasmine Arouri, Floris Jolink und Liesbeth Gunnink

1. Ich bin tätig als:
- o Arzt/Ärztin (Assistenz-, Fach-, Ober-)
- o Student(in) (PJ-, Famulant(in), Blockpraktikant(in))
- o Kranken- und Gesundheitspfleger(in) / Medizinische(r) Fachangestellte(r)
- o Andere Berufsgruppe:

Bemerkungen:

Screening

2. Ich weiß in welchen Fällen ich ein Screening auf multiresistente Bakterien ausführen muss.
   - Immer
   - Oft
   - Gelegentlich
   - Selten
   - Nie
   - Nicht zutreffend

3. Wer ist, gemäß Vereinbarung, für das Screening in der Notaufnahme/Poliklinik zuständig?
   - Arzt/Ärztin
   - Student(in)
   - Kranken- und Gesundheitspfleger(in) / Medizinische(r) Fachangestellte(r)
   - Andere Berufsgruppe:

4. Wer führt tatsächlich das Screening aus?
   - Arzt/Ärztin
   - Student(in)
   - Kranken- und Gesundheitspfleger(in) / Medizinische(r) Fachangestellte(r)
   - Andere Berufsgruppe:

5. Wenn ich Fragen zum Screening habe, dann weiß ich wo ich Informationen dazu finden kann.
   - Immer
   - Oft
   - Gelegentlich
   - Selten
   - Nie
   - Nicht zutreffend

6. Bei welchem prozentualen Anteil von aufgenommenen Patienten sind Sie, ihrer Meinung nach, in der Lage die Screening auszuführen?
   - 0%-25%
   - 25%-50%
   - 50%-75%
   - 75%-100%

7. Wenn ich das Screening nicht ausführen kann (z.B., weil der Patient zu krank ist oder ich nicht genügend Zeit habe etc.), dann finde ich das:
   - Schlecht
   - Keine Meinung
   - Gut
   - Nicht zutreffend

8. Wie zufrieden sind Sie mit der Schulung bezüglich des Screenings?
- o Sehr zufrieden
- o Ziemlich zufrieden
- o Neutral
- o Unzufrieden
- o Sehr unzufrieden
- o Nicht zutreffend

Anforderung des Screeningabstriches

9. Mir ist genau bekannt welchen Screeningabstrich ich anfordern muss.
- o Immer
- o Oft
- o Gelegentlich
- o Selten
- o Nie
- o Nicht zutreffend

10. Mir ist bekannt wie ich die Screeningabstriche in der Elektronischen Patientenakte (im Orbis® über IXSERV) anfordern muss.
- o Immer
- o Oft
- o Gelegentlich
- o Selten
- o Nie
- o Nicht zutreffend

Abnahme der Screeningabstriche

11. Mir ist genau bekannt wo und wie ich die Screeningabstriche nehmen muss.
- o Immer
- o Oft
- o Gelegentlich
- o Selten
- o Nie
- o Nicht zutreffend

12. Wenn ich einen Screeningabstrich genommen habe, weiß ich wo ich ihn hinschicken muss.
- o Immer
- o Oft
- o Gelegentlich
- o Selten
- o Nie
- o Nicht zutreffend

Folgemaßnahmen und Isolation

13. Es ist deutlich angegeben ob Isolationsmaßnahmen bei einem bestimmten Patienten erforderlich sind. Entweder im Patientenzimmer oder in der Patientenakte.
- o Immer
- o Oft
- o Gelegentlich
- o Selten
- o Nie
- o Nicht zutreffend

14. Während der Übergabe wird mir mitgeteilt welchen Krankheitserreger ein Patient hat und warum er dafür isoliert wird.
- o Immer
- o Oft
- o Gelegentlich
- o Selten
- o Nie
- o Nicht zutreffend

Compliance

15. Ich halte das Screening für gut durchführbar.
- o Immer
- o Oft
- o Gelegentlich
- o Selten
- o Nie
- o Nicht zutreffend

16. Wenn Sie Durchführen des Screenings beurteilen müssten, welche Note würden Sie erteilen?
- o Die Screening ist gut
- o Die Screening ist ausreichend
- o Die Screening ist mangelhaft
- o Die Screening ist schlecht
- o Nicht zutreffend

Verbesserungen

17. Womit sind Sie bei den aktuellen Screeningempfehlungen zufrieden?

Antwort:

18. Wie kann das Screening Ihrer Meinung nach verbessert werden?

Antwort:
